# Prevalence and risk factors of Burnout syndrome among intensive care unit members during the second wave of COVID-19: a single-center study

**DOI:** 10.31744/einstein_journal/2024AO0271

**Published:** 2024-08-20

**Authors:** Verena Laila Moniz Barreto Lima, Fernando José da Silva Ramos, Paulo Henrique Suher, Maria Aparecida Souza, Fernando Godinho Zampieri, Flavia Ribeiro Machado, Flavio Geraldo Resende de Freitas

**Affiliations:** 1 Department of Anesthesiology, Pain and Intensive Care Medicine Hospital São Paulo Universidade Federal de São Paulo São Paulo SP Brazil Department of Anesthesiology, Pain and Intensive Care Medicine, Hospital São Paulo, Universidade Federal de São Paulo, São Paulo, SP, Brazil.

**Keywords:** COVID-19, Pandemics, Intensive care units, Burnout, psychological, Stress, psychological, Anxiety, Surveys and questionnaires

## Abstract

**Objective:**

To evaluate the prevalence of burnout among the intensive care unit team of a university hospital after the second wave of COVID-19 and identify the key factors associated with its development.

**Methods:**

This single-center study included 395 employees from a multidisciplinary team. The participants completed a questionnaire based on the Maslach Burnout Inventory. Multivariate analysis was used to identify the factors associated with burnout.

**Results:**

Of 395 participants, 220 responded to the questionnaire (response rate: 56%). The prevalence of Burnout syndrome, defined as a severe score in at least one dimension, was 64.5% (142/220). Emotional distress was the most prevalent dimension, with a severe score affecting 50.5% (111/220) of the participants, followed by depersonalization at 39.1% (86/220). Only 5.9% (13/220) had severe scores in all three dimensions. Multivariate analysis revealed that being a physician was significantly associated with severe burnout symptoms in at least one dimension (odds ratio (OR), 1.32; 95% confidence interval (95%CI): 1.57-9.05; p=0.003). Additionally, having two or more jobs was associated with burnout in the three dimensions (OR=1.65; 95%CI=1.39-19.59; p=0.01).

**Conclusion:**

This study highlights the alarming prevalence of burnout among intensive care unit teams, particularly among physicians, following the second wave of COVID-19. This emphasizes the need for targeted interventions and support systems to mitigate burnout and reduce its negative impact on healthcare professionals’ well-being and patient care.

## In Brief

This single-center study highlights the high prevalence of burnout (64.5%) among 220 professionals of multidisciplinary intensive care unit teams after the second wave of COVID-19. Being a physician and having multiple jobs were prominent among the main risk factors. The results emphasize the need for specific approaches to address burnout among healthcare professionals, especially in stressful environments such as intensive care units.

## Highlights

A single-center study evaluated the prevalence and risk factors for Burnout syndrome in intensive care unit teams.

T۪he study revealed a high prevalence of Burnout syndrome in at least one dimension.

Factors such as profession and having two or more jobs were associated with severe burnout symptoms.

The study highlighted the need for interventions and support to address burnout among intensive care unit teams.

## Graphical abstract

**Figure:**
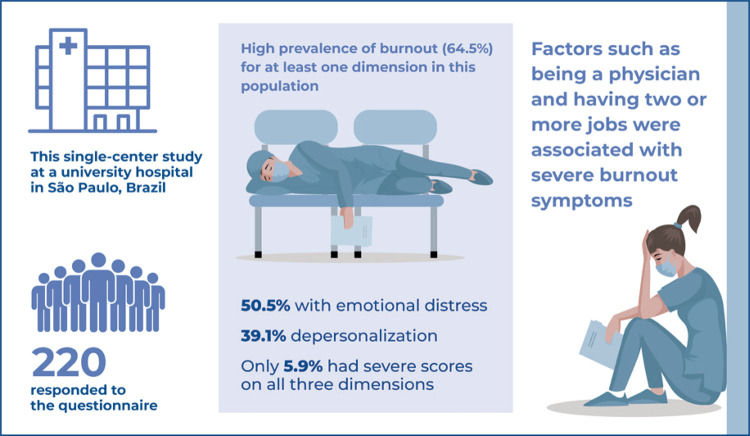

## INTRODUCTION

Stress can be defined as the response to events or situations that exceed an individual’s or social system’s capacity for adaptation or resilience.^(1)^To cope with these situations, individuals employ various strategies to promote effective adaptation. However, without adequate coping mechanisms, Burnout syndrome may develop and is characterized by psychological exhaustion and exacerbation of emotional fatigue.^(1,2)^

Maslach et al. conceptualized Burnout syndrome as a multidimensional condition with three main dimensions: emotional exhaustion, depersonalization (dehumanization), and reduced personal accomplishment in relation to work. Emotional exhaustion refers to overwhelming fatigue and the depletion of physical and mental resources that hinder an individual’s ability to perform tasks effectively. Depersonalization involves adopting negative attitudes and behaviors towards the recipients of one’s services, leading to a sense of detachment. Reduced personal accomplishment refers to a diminished sense of competence and achievement in one’s work.^(3)^

In recent years, Burnout syndrome has increased, which may be attributed to demanding work environments characterized by coldness, competition, and high stress levels, such as those found in intensive care units (ICUs).^(1,4-6)^

In December 2019, coronavirus disease 2019 (COVID-19) emerged, caused by the severe acute respiratory syndrome coronavirus 2 (SARS-CoV-2). The rapid spread of the virus has led to an unprecedented global pandemic with a significant increase in hospital admissions.^(7)^ From February 2020 to June 2021, there were over 180 million confirmed cases of COVID-19 worldwide, resulting in approximately 4 million deaths.^(8)^ This health crisis has brought about numerous stressors, both individually and collectively, particularly affecting the emotional well-being of healthcare professionals.^(2,9,10)^ Healthcare professionals, including ICU staff, are at higher risk of burnout owing to their direct exposure to infected patients and the significant responsibilities associated with patient care and safety.^(11)^

## OBJECTIVE

To evaluate the prevalence of severe symptoms of burnout in intensive care unit staff of a university hospital in São Paulo following the second wave of COVID-19 and the main factors associated with the development of this syndrome.

## METHODS

This observational cross-sectional study was conducted at a university hospital in São Paulo, Brazil. The study encompassed four distinct ICUs collectively comprising 53 beds. Among these ICUs, three were designated as medical-surgical patients and were distributed across 17, 14, and nine beds. In addition, one ICU was exclusively dedicated to neurological patients and consisted of nine beds. During the study period, all the ICUs received patients with COVID-19. The multidisciplinary team comprised 395 professionals, including 93 physicians, 69 nurses, 151 nursing assistants, 49 physiotherapists, eight psychologists, six pharmacists, six speech therapists, six nutritionists, three dentists, and four administrative assistants.

The validated Portuguese version of the Maslach Burnout Inventory (MBI) was utilized to assess Burnout syndrome.^(12)^ The MBI questionnaire consisted of 22 items distributed across three dimensions: emotional distress (ED: nine items), depersonalization (DP: five items), and professional ineffectiveness (IN: eight items). Each item is rated on a Likert-type scale ranging from “never” (zero) to “daily” (four), with respondents indicating the frequency at which they perceive or experience the statements in each question. High scores on ED and DP, combined with low scores on IN, indicate that the individual presents with Burnout syndrome, according to Maslach et al.^(3)^ Additionally, according to Grunfeld et al., Burnout syndrome can be identified by a severe score on any of the dimensions.^(13)^ A severe score was defined as ED ≥27, DP ≥13, and IN <7. This study also assessed the participants’ social and professional aspects.

The data were collected using the SurveyMonkey^®^ tool. A survey link was created and distributed to employees through a contact list via WhatsApp (^©^ 2023 Meta) and email. Additionally, QR codes with information regarding the survey were distributed in areas of coexistence within the ICUs. The survey employed a closed model requiring a password for access and ensured that each participant could submit their responses once based on their unique internet protocol address. The survey comprised 34 questions, of which 22 were specifically related to the MBI and the remaining focused on social aspects. The average time to complete the survey was estimated to be 5 minutes. The data were collected from September 30, 2021, to November 30, 2021. The contact information of the ICU collaborators, including email addresses and phone numbers, was obtained from their respective managers. Upon accessing the questionnaire, the participants were directed to a homepage containing free and informed consent forms, which needed to be completed and accepted to proceed with the research. All members of the ICU team were eligible to participate regardless of their duration of employment at the institution. Questionnaires with incomplete responses were excluded from the analysis.

The study protocol and data collection procedures were approved by the Research Ethics Committee of the *Universidade Federal de São Paulo* (CAAE: 50389921.3.0000.5505; #4.992.174), which ensured adherence to ethical standards and participant confidentiality.

The collected data were analyzed using the (SPSS) software version 20 (IBM, Armonk, NY, USA). Categorical variables were summarized using absolute and relative frequencies, whereas quantitative variables were described using measures of central tendency (mean and median) and dispersion when appropriate.

To assess the factors associated with Burnout syndrome, a χ^2^ test or Fisher’s exact test was used for nominal data. Furthermore, backward logistic regression was performed to identify the factors independently associated with the development of severe burnout symptoms. All variables with a p<0.20 in the univariate analysis were included in the model. For the logistic regression analysis, the professions were grouped into three categories: physicians, nursing (nurses and nursing assistants), and others (*e.g*., physiotherapist, psychologist, pharmacist), with the others serving as the reference. The duration of employment at the institution was categorized into two groups: up to 7 years and over 7 years, and the number of jobs was categorized as having only one job or having two or more jobs. Additionally, the number of working hours per week was divided into two groups: up to 60 hours per week and more than 60 hours per week. The association between variables was estimated using odds ratios (OR) and their respective 95% confidence intervals (95%CI). Statistical significance was set at p<0.05.

## RESULTS

**Figure 1:**
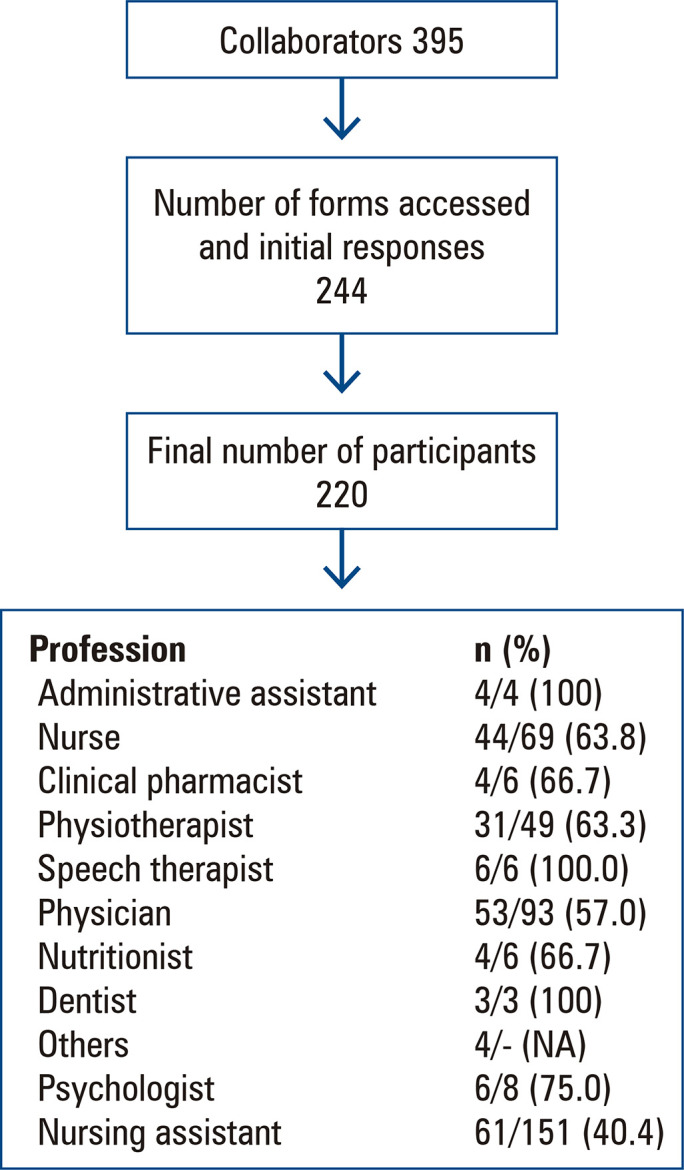
Selection process of participants

The study sample included 220 respondents, accounting for 56% of the multidisciplinary teams working in the ICU (Figure 1). The largest number of participants were nursing assistants, comprising 61 individuals (27.7%), followed by physicians with 53 (24.1%). We obtained participation from all invited professionals. Table 1 provides an overview of the participants’ characteristics. Most participants were female (76.8%, n=169). The predominant age group was 26-35 years old, followed by 36-45 years old. More than 50% reported working in their current profession for up to 6 years.

**Table 1:** Characteristics of the participants

Characteristics	
Profession	
Administrative assistant	4 (1.8)
Nurse	44 (20.0)
Clinical pharmacist	4 (1.8)
Physiotherapist	31 (14.1)
Speech therapist	6 (2.7)
Physician	53 (24.1)
Nutritionist	4 (1.8)
Dentist	3 (1.4)
Others	4 (1.8)
Psychologist	6 (2.7)
Nursing assistant	61 (27.7)
Sex	
Male	49 (22.2)
Female	169 (76.8)
Prefer not to answer	2 (1.0)
Age	
16-25 years	49 (22.3)
26-35 years	74 (33.6)
36- 45 years	73 (33.2)
46-55 years	23 (10.5)
56- 65 years	1 (0.5)
>65 years	0
Current resident at *Hospital São Paulo*?	
Yes	58 (26.4)
No	158 (71.8)
Prefer not to answer	4 (1.8)
How long have you been practicing your profession?	
0-3 years	84 (38.2)
4-6 years	31 (14.1)
7-10 years	27 (12.3)
11-14 years	29 (13.2)
>15 years	49 (22.3)
How long have you worked at Hospital São Paulo?	
<1 year	58 (26.4)
1-3 years	82 (37.3)
4-7 years	26 (11.8)
8-10 years	20 (9.1)
10-15 years	13 (5.9)
>15 years	21 (9.5)
How many jobs do you have?	
Only this	131 (59.5)
I work in 2 jobs	63 (28.6)
Work 3 or more jobs	26 (11.9)
What are your current weekly working hours?	
<40 hours	72 (32.7)
41-60 hours	103 (46.8)
61-80 hours	34 (15.5)
>80 hours	11 (5.0)
What are your main working hours?	
Daytime	171 (77.7)
Night	40 (40.1)
I don’t have a fixed time	9 (18.2)
Are you married or have a stable relationship?	
Yes	107 (48.6)
No	113 (51.4)
Do you have children?	
Yes	80 (36.4)
No	140 (63.6)

Results in number (%).

The prevalence of Burnout syndrome was 64.5% (142/220). The dimension with the highest prevalence of severe scores was ED (111/220, 50.5%), followed by DP (86/220, 39.1%). Among professional categories, nutritionists had the highest prevalence of burnout symptoms in one dimension, with all four participants (100%) exhibiting severe scores. Physicians and nurses had notable prevalence rates of 83% (44/53) and 72.7% (32/44), respectively. Only 13 participants (5.9%) had severe scores on all three dimensions (Table 2). Physicians had the highest prevalence of severe scores in all three dimensions, with seven participants (13%, 7/53).

**Table 2:** Frequency of severe scores in each dimension and prevalence of Burnout syndrome

Dimensions	Severe score	

	Yes n (%)	No n (%)
Emotional distress	111 (50.5)	109 (49.5)
Depersonalization	86 (39.1)	134 (60.9)
Professional ineffectiveness	28 (12.7)	192 (87.3)
Burnout syndrome		
1 dimension	142 (64.5)	
3 dimensions	13 (5.9)	

Results in numbers (%).

Univariate analysis revealed that profession, length of service at the institution, number of jobs, and weekly working hours were factors associated with severe scores on at least one dimension of Burnout syndrome (Table 3). However, only the number of jobs showed a statistically significant association with severe scores in all three dimensions (p=0.01).

**Table 3:** Factors related to severe scores on a burnout dimension – univariate analysis

	Burnout syndrome		All (220) n (%)	p value

	No (78) n (%)	Yes (142) n (%)		
Profession				0.003
Administrative assistant	1 (25.0)	3 (75.0)	4 (1.8)	
Nurse	12 (27.3)	32 (72.7)	44 (20.0)	
Clinical pharmacist	2 (50.0)	2 (50.0)	4 (1.8)	
Physiotherapist	13 (41.9)	18 (58.1)	31 (14.1)	
Speech therapist	2 (33.3)	4 (66.7)	6 (2.7)	
Physician	9 (16.9)	44 (83,1)	53 (24,1)	
Nutritionist	0 (14)	4 (100.0)	4 (1.8)	
Dentist	3 (100.0)	0 (14)	3 (1.4)	
Other	3 (75.0)	1 (25.0)	4 (1.8)	
Psychologist	3 (50.0)	3 (50.0)	6 (2.7)	
Nursing assistant	30 (49.2)	31 (50.8)	61 (27.7)	
Sex				0.09
Female	66 (39.0)	103 (61.0)	169 (76.8)	
Male	12 (24.4)	37 (75.6)	49 (22.3)	
Prefer not to answer	0 (14)	2(100.0)	2 (0.9)	
What is your age group?				0.40
16-25 years	19 (38.8)	30 (61.2)	49 (22.3)	
26-35 years	21 (28.4)	53 (71.6)	74 (33.6)	
36-45 years	27 (36.9)	46 (63.1)	73 (33.2)	
46-55 years	11 (47.8)	12 (52.2)	23 (10.5)	
56-65 years	0 (14)	1 (100.0)	1 (0.5)	
Are you currently resident at the institution?				0.21
No	60 (38.0)	98 (62.0)	158 (71.8)	
Yes	18 (31.0)	40 (69.0)	58 (26.4)	
Prefer not answer	0 (14)	4 (100.0)	4 (1.8)	
How long have you been practicing your profession?				0.86
0-3 years	32 (38.1)	52 (61.9)	84 (38.2)	
4-6 years	10 (32.2)	21 (67.8)	31 (14.1)	
7-10 years	8 (29.6)	19 (70.4)	27 (12.3)	
11-14 years	9 (31.0)	20 (69.0)	29 (13.2)	
≥15 years	19 (38.8)	30 (61.2)	49 (22.3)	
How long have you worked at *Hospital São Paulo*?				0.01
<1 year	27 (46.5)	31 (53.4)	58 (26.4)	
1-3 years	27 (32.9)	55 (67.1)	82 (37.3)	
4-7 years	8 (30.8)	18 (69.2)	26 (11.8)	
8-10 years	3 (15.0)	17 (85.0)	20 (9.1)	
11-15 years	1 (7.7)	12 (92.3)	13 (5.9)	
>15 years	12 (57.1)	9 (42.9)	21 (9.5)	
How many jobs do you have?				0.02
Only this job	52 (39.7)	79 (60.3)	131 (59.5)	
I work in two companies	23 (36.5)	40 (63.5)	63 (28.6)	
Work in three or more companies	3 (11.5)	23 (88.5)	26 (11.8)	
What are your current weekly working hours?				0.03
<40 hours per week	34 (47.2)	38 (52.8)	72 (327)	
41-60 hours per week	34 (33.0)	69 (67.0)	103 (46.8)	
61-80 hours per week	9 (26.5)	25 (73.5)	34 (15.5)	
>80 hours per week	1 (0.9)	10 (90.1)	11 (5.0)	
What are your main working hours?				0.32
Daytime	57 (33.3)	114 (66.6)	171 (77.7)	
Night	16 (40.0)	24 (60.0)	40 (18.2)	
I don’t have a fixed time	5 (55.5)	4 (45.5)	9 (4.1)	
Are you married or have a stable relationship?				0.78
No	39 (34.5)	74 (65.5)	113 (51.4)	
Yes	39 (36.4)	68 (63.5)	107 (48.6)	
Do you have children?				0.66
No	48 (34.3)	92 (65.7)	140 (63.6)	
Yes	30 (37.5)	50 (62.2)	80 (36.4)	

ICU: intensive care unit.

Further analysis using logistic regression demonstrated that being a physician was independently associated with severe burnout symptoms development in at least one dimension (OR= 1.32; 95%CI= 1.57-9.05; p=0.003).Additionally, having two or more jobs was associated with severe symptom development in the three dimensions (OR= 1.65; 95%CI= 1.39-19.59; p=0.01). Physicians had the highest proportion of multiple jobs (96.2%, 51/53), followed by physiotherapists (41.9%, 13/31).

## DISCUSSION

Our study revealed a significantly high prevalence of burnout symptoms among multidisciplinary ICU teams in the aftermath of the second wave of the COVID-19 pandemic. Specifically, being a physician was associated with severe scores on at least one dimension of burnout, whereas having multiple jobs was linked to burnout symptoms across all three dimensions.

Burnout syndrome has been extensively studied in the context of ICU healthcare workers, with physicians and nursing staff particularly susceptible to its effects.^(4,5,14,15)^ Pre-pandemic studies in Europe reported a burnout prevalence of up to 45% among physicians and nurses, considering three dimensions.^(5,16)^However, studies conducted in Brazil reported contradictory results. Barros et al. reported a prevalence of 63.3% in one dimension and 7.4% in all three dimensions of burnout among intensivists in Salvador, Bahia.^(14)^Similar results were reported by Tironi et al. in a study assessing burnout prevalence among intensive care physicians in five Brazilian capitals using the MBI.^(11)^A Brazilian study conducted in a private ICU before the pandemic identified high levels of severe burnout across all three dimensions: 18% for physicians, 25% for physiotherapists, and nearly 35% for intensive care nurses.^(6)^In contrast, Alvares et al. assessed burnout symptoms in 17 public ICUs in Brazil and discovered a prevalence of only 0.41% for severe symptoms across all three dimensions but 36.9% for symptoms in one dimension.^(17)^

Available data on the pandemic indicate a heightened prevalence of Burnout syndrome. Factors such as the highly contagious nature of COVID-19, occupational risks, resource scarcity, and continuous exposure of healthcare workers on the front lines contribute to the development of severe burnout.^(2,9)^A systematic review of 13 studies revealed a pooled prevalence of 23.2% for anxiety and 22.8% for depression.^(18)^In a study conducted in Italy involving 376 healthcare professionals, more than one-third exhibited high levels of ED, one-fourth reported elevated DP, and approximately 15% experienced low levels of IN.^(19)^Azoulay et al. documented a prevalence of 51% severe burnout among intensivists during the first wave of the COVID-19 pandemic.^(20)^More recently, Fumis et al. examined intensive care physicians at a private hospital in São Paulo, Brazil and found a burnout prevalence of 96.1% when considering the presence of at least one dimension and 37.2% for severe scores across all three dimensions. The study reported a 50% increase in the prevalence of burnout among medical teams compared with the pre-pandemic period.^(21)^In our study, we observed a high prevalence of burnout among physicians, with 83% experiencing symptoms in at least one dimension and 13% exhibiting symptoms across all three dimensions.

A recent meta-analysis of burnout in physicians and nurses working in adult ICUs revealed a prevalence of over 40% among all professionals. The authors found a nonconsensual definition of high-level Burnout syndrome using the MBI. For ICU physicians, no difference in burnout prevalence between the COVID-19 pandemic and pre-pandemic periods was observed. However, for nurses, the prevalence of high levels of burnout was higher during the pandemic than before the COVID-19 pandemic period. A subgroup analysis based on country income reported that burnout showed no differences between high- and upper-middle-income countries.^(22)^

In this study, we examined various factors associated with the development of severe burnout in at least one dimension. However, upon conducting multivariate analysis, we observed that only the physician profession was significantly related to the development of burnout. This finding suggests that being a physician may increase burnout risk. One possible explanation for this association is the higher workload and the presence of multiple job responsibilities that physicians often face. Previous research has established that working hours and the number of jobs are significant factors linked to burnout, and these aspects can be challenging to manage owing to socioeconomic considerations that extend beyond the work environment.^(16)^

Although our study was conducted at a single center, we included a multidisciplinary team that allowed us to examine the presence of burnout among various professionals, including nutritionists and clinical pharmacists. Data was collected anonymously to ensure participants’ confidentiality and enhance our results’ internal validity. We achieved a high response rate, further strengthening our findings’ reliability. By employing an internationally validated questionnaire, we enable comparisons with previously published data, facilitating a comprehensive understanding of burnout in the context of our study. Additionally, we explored the profiles of employees, which can contribute to the development of targeted interventions to promote the overall well-being and health of multidisciplinary ICU teams.

Our study had some limitations. First, it was conducted during the final stages of the second wave of the COVID-19 pandemic, and it is possible that despite exhibiting symptoms of burnout, professionals may have experienced a sense of hope and resilience with the approaching end of the second wave, thus minimizing the findings. Second, owing to the study’s observational design, we could only establish associations between variables and not causality. Third, our study did not allow for an in-depth assessment of the characteristics of professionals with severe burnout symptoms. Emotional distress and DP are the main dimensions related to the development of burnout; however, depression and anxiety may also play a role.^(23)^

## CONCLUSION

Our findings indicate a high prevalence of severe burnout symptoms among multidisciplinary intensive care units teams during the second wave of the COVID-19 pandemic, significantly impacting healthcare professionals. Profession, particularly being a physician, along with the number of jobs and working hours, were associated with burnout symptoms development in at least one dimension. Moreover, the number of jobs was specifically associated with the presence of burnout symptoms in all three dimensions. These results highlight the urgent need for targeted interventions and support mechanisms to address burnout among healthcare professionals, particularly in high-stress environments such as intensive care units.
